# Pharmacokinetics and Exposure–Response During Infliximab Induction Therapy in Pediatric IBD Using Point-of-Care Assay

**DOI:** 10.3390/jcm14227968

**Published:** 2025-11-10

**Authors:** Amy Hemperly, Jincheng Yang, Anh Ta, Niels Vande Casteele

**Affiliations:** 1 Department of Pediatric Gastroenterology, University of California San Diego, La Jolla, San Diego, CA 92093, USA; amy.hemperly@gmail.com; 2 Skaggs School of Pharmacy and Pharmaceutical Science, University of California San Diego, La Jolla, San Diego, CA 92093, USA; jinchengyang2@gmail.com (J.Y.); anh3011@gmail.com (A.T.); 3 Department of Medicine, University of California San Diego, La Jolla, San Diego, CA 92093, USA

**Keywords:** pediatric, infliximab, pharmacokinetic model

## Abstract

**Background**: Pharmacokinetic therapeutic failure with infliximab during induction therapy can pose a significant challenge for clinicians. The objective of this study was to conduct population pharmacokinetic and exposure–response analyses in children with inflammatory bowel disease during infliximab induction therapy. **Methods**: A prospective single-center observational study was conducted in anti-TNF naïve pediatric patients < 21 years of age with IBD starting infliximab according to their physicians’ clinical judgment between December 2018 and December 2020. Population pharmacokinetic analysis was conducted by nonlinear mixed-effects modeling using infliximab serum levels measured by RIDASCREEN^®^ enzyme-linked immunosorbent assay. Infliximab serum levels were also measured by point-of-care (POC) assay using RIDA^®^QUICK IFX monitoring and the RIDA^®^QUICK SCAN II. An exposure–response analysis was conducted to evaluate the association between infliximab concentrations and efficacy outcomes at week 14. **Results**: The typical value of infliximab clearance in a pediatric patient with IBD weighing 51 kg was 0.252 L/day, and the Vc was 3.43 L and Vp was 2.11 L. Weight and albumin were identified to be significant covariates on clearance in the final model. Tertile analysis of infliximab exposure showed an exposure–response relationship in which higher infliximab ELISA concentrations during induction therapy were associated with clinical remission at week 14 and biochemical response at week 14, but the trend did not reach statistical significance due to the small sample size. The concordance correlation coefficient between the infliximab ELISA and the POC assay was 0.905 [0.867, 0.933]. **Conclusions**: We report parameter estimates during infliximab induction therapy in pediatric patients with inflammatory bowel disease. Weight and albumin were identified to be significant covariates on clearance. ELISA and POC infliximab assays showed comparable results, supporting the role of POC testing for real-time therapeutic drug monitoring.

## 1. Introduction

Infliximab is an effective treatment for patients with moderate to severe pediatric inflammatory bowel disease (IBD). However, up to 30% of patients with pediatric IBD do not respond to initial therapy with infliximab [1,2]. Many cases of therapeutic failure can be attributed to low serum drug trough concentrations at the end of induction [3].

Higher infliximab concentrations during induction therapy are associated with short- and long-term favorable clinical and endoscopic outcomes [4,5,6].

Proactive therapeutic drug monitoring can be used to tailor dosing and optimize infliximab concentrations during induction [7,8]. However, commercial infliximab assays may have delayed turnaround times, making results unavailable in time to influence clinical decisions. Model-informed therapeutic drug monitoring with a point-of-care infliximab assay could inform dosing to reduce variability in exposure during induction therapy.

The aims of this prospective study were to develop a population pharmacokinetic model using serum samples from pediatric subjects with IBD initiating infliximab, identify covariates contributing to variability in infliximab clearance and volume of distribution, and apply the model to simulate various measures of exposure for correlation with clinical outcomes. Additionally, we aimed to compare a novel point-of-care assay (POC) for rapid infliximab quantification to the enzyme-linked immunosorbent assay (ELISA), which is the conventional assay used for therapeutic drug monitoring of infliximab. We hypothesized that inter-individual variability in infliximab exposure, partly driven by accelerated clearance, affects clinical outcomes in pediatric IBD patients and that the POC assay was comparable to ELISA.

## 2. Materials and Methods

### 2.1. Study Design

A prospective single-center observational study was conducted in anti-TNF naïve pediatric patients with IBD starting infliximab to develop a population pharmacokinetic model during induction therapy. Patients from 6 years of age to 21 years of age with active inflammatory bowel disease, as confirmed by endoscopic evaluation, who were initiating infliximab between December 2018 and December 2020 were eligible to enroll. Enrolled participants were treated with infliximab according to physician judgment. The weighted Pediatric Crohn’s Disease Activity Index (wPCDAI) for patients with Crohn’s disease (CD) or the Pediatric Ulcerative Colitis Activity Index (PUCAI) for patients with ulcerative colitis (UC) or IBD-U (inflammatory bowel disease-unclassified) was collected prior to infliximab infusions at weeks 0 and 12. Serum was collected prior to all infliximab infusions during induction for infliximab quantification by a point-of-care lateral flow-based assay and infliximab and antibody-to-infliximab concentrations by ELISA. Serum was also obtained approximately one hour after completion of the first and second infusions for an infliximab peak concentration. In patients who were hospitalized, serum was collected at the same time as blood collection for routine clinical care for an infliximab intermediate concentration. Stool samples were collected at weeks 2, 6, and 14. Clinical and laboratory data were collected from their medical records for one year.

Study protocol and materials were approved by the institutional review board at the University of California San Diego (UCSD). All patients provided written informed consent.

### 2.2. Outcomes

Clinical response, the main efficacy outcome for the study, was defined as a decrease from baseline wPCDAI of >17.5 points for CD or a decrease from baseline PUCAI of >20 points for UC or IBD-U. Clinical remission was defined by a wPCDAI < 12.5 for CD or a PUCAI < 10 for UC or IBU-U. Biochemical response was defined by a 50% percent decrease in serum C-reactive protein (CRP) or fecal calprotectin if elevated at baseline (CRP > 0.5 mg/L or fecal calprotectin > 250 μg/g). Biochemical remission was defined by normalization of CRP (<0.5 mg/L) or fecal calprotectin (<250 μg/g) in patients with elevated biomarker concentrations at baseline. The patient met the criteria for primary remission post-induction if the patient met the criteria for clinical and biochemical remission. Durability of infliximab use was defined as continued infliximab use at one year after infliximab initiation.

### 2.3. Measurements

#### 2.3.1. Enzyme-Linked Immunosorbent Assay

Serum infliximab and anti-infliximab antibody measurements were performed by RIDASCREEN^®^ ELISA (R-Biopharm, Darmstadt, Germany) according to the manufacturer’s instructions. The limit of infliximab detection was 27.3 ng/mL. Anti-infliximab antibody concentrations in the setting of a subtherapeutic infliximab concentration (<1 μg/mL) were considered clinically significant. The limit of anti-infliximab antibody detection was 0.62 ng/mL at 1:25 dilution and 5.04 ng/mL at 1:200 dilution.

#### 2.3.2. Point-of-Care Assay

POC infliximab measurements were performed using RIDA^®^QUICK IFX monitoring and a RIDA^®^QUICK SCAN II (R-Biopharm, Darmstadt, Germany). The test is a lateral flow immunochromatographic assay for the quantitative detection of infliximab in serum and plasma. Infliximab was detected through the formation of an antibody–antigen-sandwich made visible by the usage of marked colloidal gold nanoparticles. The generated signal was read out with the RIDA^®^QUICK SCAN II and the infliximab concentration was calculated by using the standard curve which is stored in the instrument. The assay was carried out in a clinical research laboratory. The limit of detection and quantification was <0.5 µg/mL. The device was used for research purposes only and clinical decisions were not based on the result.

### 2.4. Population PK Mode Development

Sparse infliximab serum levels measured with ELISA were analyzed using a nonlinear mixed-effects modeling software, NONMEM^®^ version 7.3 (Gaithersburg LLC, Ellicott City, MD, USA), following a prospectively planned evaluation to develop a population pharmacokinetic model for infliximab in pediatric IBD patients. The structural pharmacokinetic model component, which estimates the pharmacokinetic parameters to describe the concentration–time profile of infliximab, was established. This stochastic model consisted of exponential-normal distribution to capture interindividual variabilities, and a proportional error was used to describe any residual sources of variabilities. The following baseline covariates were evaluated in the covariate model: sex, race, ethnicity, diagnosis, weight, body mass index (BMI), age at infliximab start, hemoglobin, albumin, CRP, erythrocyte sedimentation rate (ESR), white-blood-cell count (WBC), disease activity index, corticosteroid use, concomitant immunomodulator use, and infliximab dose (mg/kg). Structural pharmacokinetic parameters were scaled by size prior to covariate assessments. A modeling covariate approach was used to evaluate the association between patient characteristics and PK parameters. Covariate screening was conducted in a stepwise approach in which potential covariates were first added univariately onto the model. Covariates that improve the model fit by a change in objective function value (OFV) of at least 3.84 (*p* < 0.05) were retained following the initial covariate screening. Covariates identified as significant were evaluated in a stepwise forward selection approach and covariates which improved the OFV by 7.88 (*p* < 0.005) were retained in the final model. The models during covariate evaluation were accepted if they converged with a successful covariance step. The population pharmacokinetic models were assessed for appropriateness using conventional criteria, including convergence status, likelihood ratio test, parameter precision, assessment of goodness-of-fit plots, and shrinkage. In addition, the final population model was assessed using a bootstrap validation method and graphically through a visual predictive check.

### 2.5. Exposure–Response Analysis

The primary focus was the association between infliximab concentration and efficacy outcomes at week 14 (time point for induction efficacy). The prognostic values of earlier infliximab concentrations on subsequent efficacy outcomes were also evaluated. Exploratory evaluations were carried out to graphically evaluate associations between the simulated metrics of exposure and response to therapy. Figures were faceted on likely predictive factors (e.g., sex, age, disease characteristics, etc.).

### 2.6. Statistical Analysis

For the population pharmacokinetic analysis, no formal sample size calculation was performed. From previous studies, it is expected that a convenience sample of 30 patients with intense serum sampling would be sufficient to test for covariates that impact drug exposure and evaluate exposure–response [9].

Infliximab concentrations were compared between patients achieving and not achieving the prespecified efficacy outcomes using a Wilcoxon two-sample test. The effects of covariates that may influence the exposure–response relationship during induction and maintenance therapy were assessed by univariable logistic regression modeling. In a tertile analysis, the proportion of patients achieving the efficacy outcome per infliximab concentration tertile (33%, 66%, 99%) were compared using Fisher’s exact test and a one-sided exact Cochran–Armitage test. Statistical analyses of the data and figures produced were performed using SPSS (V28). Numerical variables were summarized by median and interquartile range (IQR) and categorical variables were summarized by frequency and percent. The Chi-squared test was used for univariate analysis of discrete variables and the Mann–Whitney U test was used for univariate analysis of continuous variables. Agreement between the POC and ELISA was demonstrated on a Bland–Altman plot with a 95% of limit of agreement and confidence interval. A concordance correlation coefficient was calculated. R 4.0.2 was used for all analyses and a *p*-value of <0.05 was considered statistically significant.

## 3. Results

### 3.1. Patient Characteristics

Thirty-one patients were enrolled and one patient withdrew from the study. Demographic and clinical characteristics are shown in Table 1. Of the 30 patients that completed the study, 17 (57%) had CD, 7 (23%) had UC, and 6 (20%) had IBD-U. The median age was 15 years (8–17) and the median weight was 51.2 kg (24.9–92.8). Two patients had mild malnutrition, four patients had moderate malnutrition, and one patient had severe malnutrition as classified using BMI-for-age z-score. The majority of patients had extensive disease and 17 patients (57%) were hospitalized at the time of infliximab induction. All patients were biologic naïve. The baseline median wPCDAI score in patients with CD was 41 (IQR 30–62) and the median PUCAI score was 50 (IQR 30–70) in patients with UC or IBD-U. Twelve patients were on corticosteroids (40%) and thirteen patients (43%) were on a concomitant immunomodulator. Of the thirteen patients on a concomitant immunomodulator, nine were on methotrexate and four were on a thiopurine.

### 3.2. Patient Outcomes: Clinical and Biochemical Response/Remission

Efficacy outcomes are shown in Table 2. Six patients (*n* = 29, 20%) met the criteria for primary remission after induction. Nineteen patients (63%) were in clinical response and ten patients (33%) were in clinical remission at week 14. Fifteen patients (*n* = 26, 58%) were in biochemical response and eight (*n* = 25, 32%) were in biochemical remission at week 14. Twenty-five patients (83%) continued on infliximab one year after initiation. None of the patients had detectable antibodies to infliximab during induction therapy using the drug-sensitive ELISA.

### 3.3. Concordance Correlation Coefficient Between ELISA and Point-of-Care Assay

**Figure 1:**
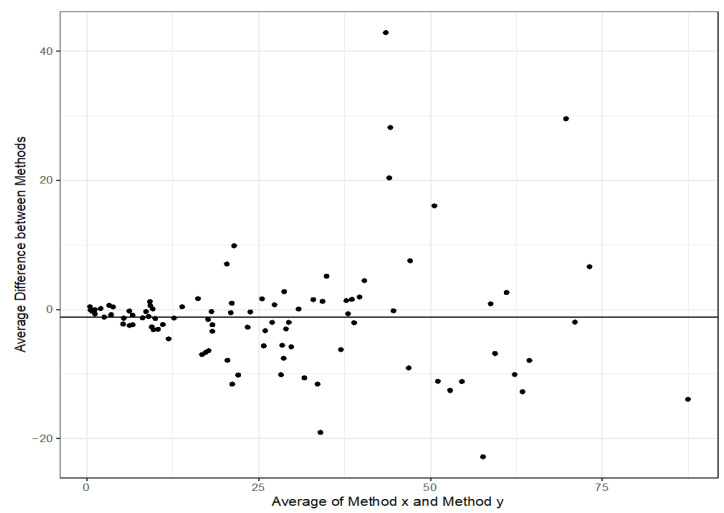
Bland–Altman plot of infliximab levels (mg/L) to compare the correlation between the ELISA and the POC assay (RIDAQUICK). The middle of the orange bar represents the 95% limit of agreement and the orange bars represent the 95% CI for limit of agreement.

Quantitative comparison showed a good correlation between the ELISA and the POC assay (RIDAQUICK) (Figure 1). The concordance correlation coefficient (CCC) was 0.905 [0.867, 0.933].

### 3.4. Population PK Modeling

**Figure 2:**
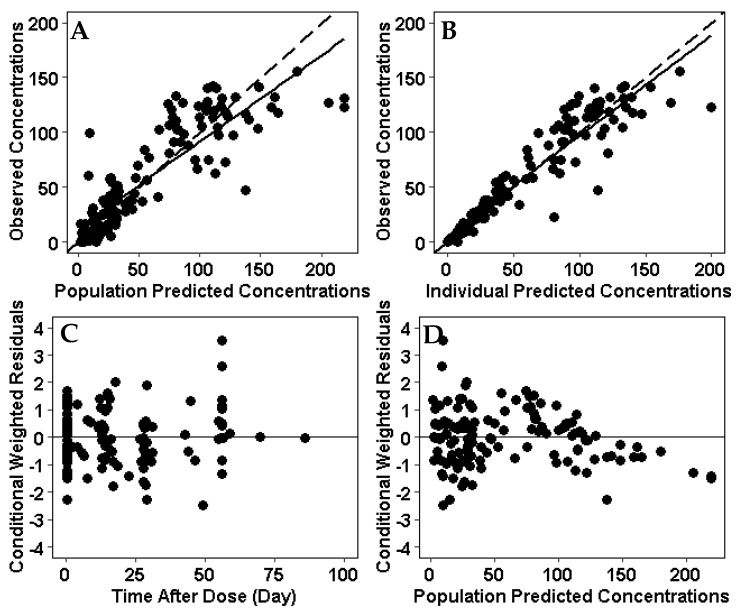

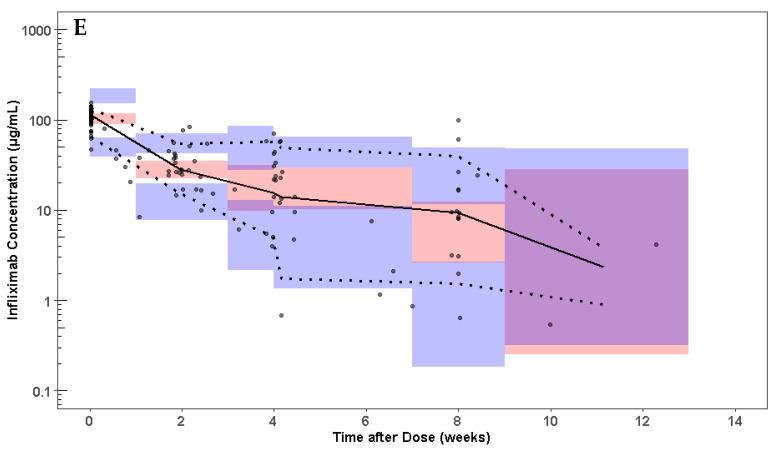
Goodness of fit plots for the infliximab population PK model. (**A**) Observed concentrations were compared with population predictions from the population PK model. (**B**) Observed concentrations were compared with individual predicted concentration. (**C**) Conditional weighted residual for each sample compared to time after dose. (**D**) Conditional weighted residuals compared with population predictions from the population PK model. (**E**) Visual predictive check of the infliximab dose over time after dose. Solid line and dotted lines represent the median and 10th and 90th percentile of the observed data, respectively. The inner red shaded area represents the 95% confidence interval around the predicted median. The outer blue shaded area represents the upper 97.5% and lower 2.5% of the 80% prediction interval. The model represents the observed infliximab concentrations without bias.

A two-compartment model was used as the structural model for the population pharmacokinetic analysis. The population pharmacokinetic parameters estimated from the final model in a pediatric patient with IBD weighing 51 kg were as follows (typical value ± standard error): clearance (CL), 0.252 ± 0.024 L/day; central compartment volume of distribution (Vc), 3.43 ± 0.22 L; peripheral compartment volume of distribution (Vp), 2.11 ± 0.40 L; and intercompartmental clearance (Q), 1.41 ± 0.44 L/day (Table 3). The median terminal half-life for infliximab was 15 days. Goodness-of-fit and visual predictive check plots obtained from the final model show that the predicted values match the measured values and that there is no time-dependent bias in the model (Figure 2).

### 3.5. Relationship of Covariates and Model Parameters

**Figure 3:**
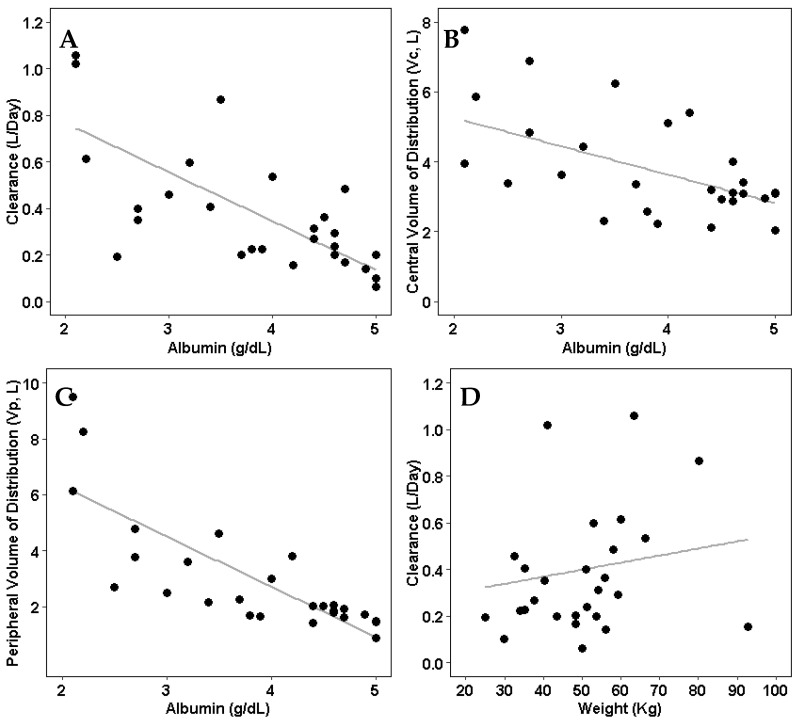
Individual pharmacokinetic parameters compared to significant covariates (albumin and weight). (**A**) Model-estimated clearance compared with patients’ albumin levels. (**B**) Model-estimated central volume of distribution compared with patients’ albumin levels. (**C**) Model-estimated peripheral volume of distribution compared with patients’ albumin levels. (**D**) Model-estimated clearance compared with patients’ weight.

Weight and albumin were identified to be significant in the final model. Both clearance and volume of distribution were inversely correlated with patients’ baseline albumin levels (Figure 3).

### 3.6. Exposure–Response Relationship

**Figure 4:**
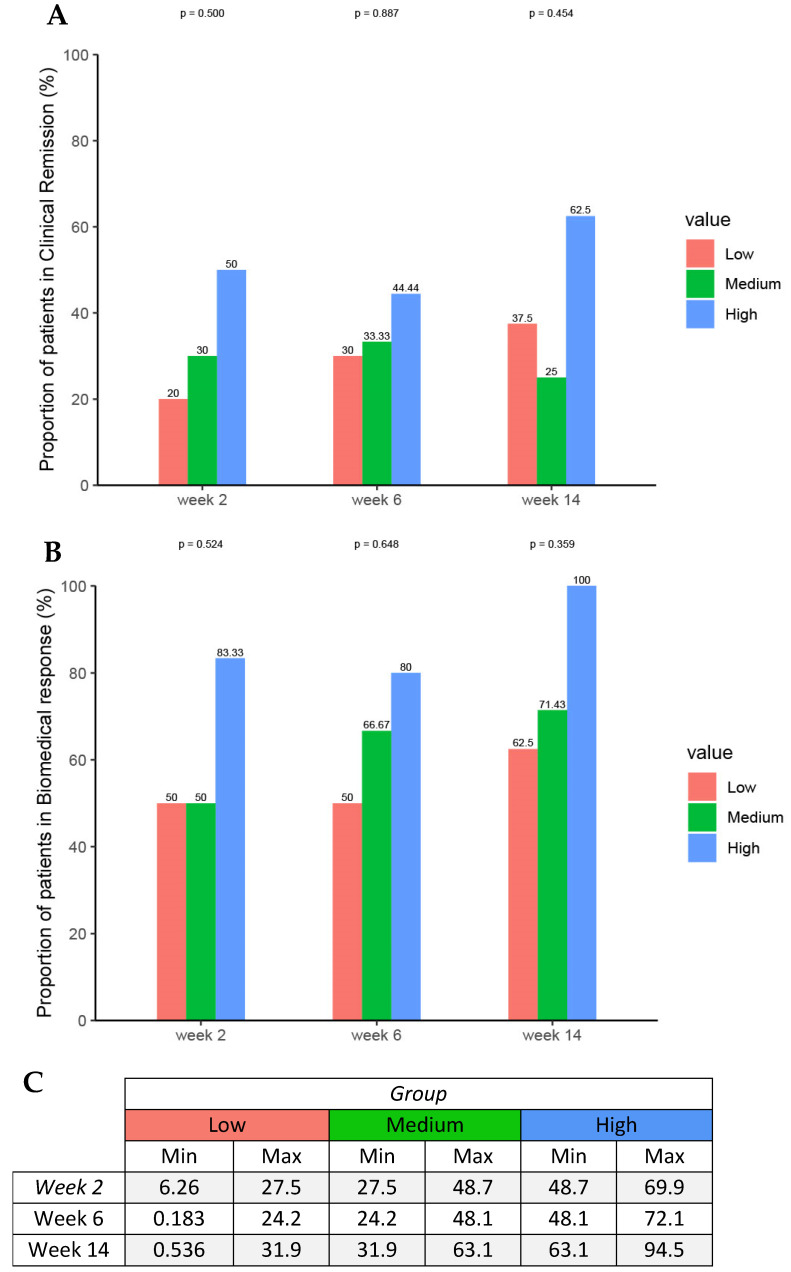
Proportions of patients achieving (**A**) clinical remission and (**B**) biochemical response by (**C**) serum infliximab concentration tertiles at week 2, 6, and 14 using Fisher’s exact test and the one-sided exact Cochran–Armitage test.

Four patients (13%) received an accelerated infliximab dosing regimen. Median infliximab concentrations were similar between pediatric patients who achieved clinical response (10 μg/mL; IQR 4–17 μg/mL) and pediatric patients who did not achieve clinical response (10 μg/mL, IQR 5–12 μg/mL) at Week 14. There was no relationship between infliximab concentrations at Week 2, Week 6, Week 14, and cumulative absolute dose during induction and efficacy outcomes at Week 14. Tertile analysis of infliximab exposure showed an exposure–response relationship in which higher infliximab ELISA concentrations during induction therapy were associated with clinical remission at week 14 and biochemical response at week 14. The trend did not reach statistical significance due to the small sample size (Figure 4).

## 4. Discussion

In this single-center study, we conducted population pharmacokinetic and exposure–response analyses from data collected prospectively during infliximab induction therapy in pediatric patients with IBD. The typical value of infliximab clearance in a pediatric patient with IBD weighing 51 kg was 0.252 L/day, and the Vc was 3.43 L and Vp was 2.11 L. These parameter estimates are comparable to previously published infliximab population PK models in pediatric IBD (Table 4). Consistent with findings of infliximab and some other biologics, weight and albumin were identified to be significant covariates on clearance in the final model. The effects of antidrug antibodies on clearance could not be assessed because no patients were positive for antibodies to infliximab during induction therapy. This finding may be attributed to the use of a drug-sensitive ELISA and the inability of the assay to detect ADA in the presence of a defined concentration of drug, the small sample size, the ability of clinicians to optimize treatment with accelerated dosing, and the time it takes for antidrug antibody development.

Results of the exposure–response analysis did not indicate that higher exposure at Week 2 and Week 6 would yield clinically meaningful improvements in efficacy at Week 14. Nevertheless, a positive exposure–response trend was observed, which is in line with other studies that have demonstrated that optimized infliximab induction is associated with long-term efficacy outcomes and sustained durable remission [14,15]. Due to the small sample size, caution should be exercised in generalizing these findings and larger, prospective studies are needed to confirm these observations.

Clinical response and clinical remission rates at the end of induction were lower than anticipated, which may be due to selection bias. Approximately 10% of patients received an accelerated dosing regimen and approximately 60% of patients were hospitalized at the time of infliximab initiation. It is in these setting where POC assays could improve real-time infliximab quantification over ELISAs. Different techniques are used to measure infliximab concentrations, including enzyme-linked immunosorbent assay (ELISA), the high-pressure liquid chromatography-based homogeneous mobility shift assay (HMSA), and the electrochemiluminescence-based immunoassay (ECLIA), but results are not immediately available to direct management in real-time. The expected turnaround time may be days to a week when the specimen needs to be sent-out to a diagnostic lab. A POC assay for infliximab quantification allows for the immediate adjustment of infliximab dosage to maintain optimal infliximab drug exposure. The infliximab-quantification system used here was a bench top-sized reader which can be operated by any nurse, technician, or physician without the requirement of specific laboratory facilities, and it had a turnaround time of 20 min. Our results showed good correlation between the ELISA and the POC assay (RIDAQUICK) and suggest that RIDAQUICK can be a practical alternative for therapeutic drug monitoring to inform clinical care and personalize treatment for pediatric patients with IBD when properly validated for its intended use.

## Figures and Tables

**Table 1:** Baseline demographics and disease characteristics.

Sex, *n* (%) Male Female	17 (57) 13 (43)
Race, *n* (%) White Asian Black or African American Native Hawaiian or Other Pacific Islander American Indian or Alaska Native Other	20 (67) 4 (13) 0 (0) 1 (3) 0 (0) 5 (17)
Ethnicity Not Hispanic or Latino Hispanic or Latino	20 (67) 10 (33)
Diagnosis, *n* (%) CD UC IBDU	17 (57) 7 (23) 6 (20)
Age at infliximab start, years, median (range)	15 (8–17)
Body weight, kg, median (range)	51.2 (24.9–92.8)
BMI, z-score, median (range)	0.1 (−3.2–1.8)
Duration of disease, years, median (range)	1 (0–10)
UC location, *n* (%) Proctitis Left Sided Pancolitis	0 (0) 1 (3) 6 (20)
CD location, *n* (%) Ileal Colonic Ileocolonic Upper GI	2 (7) 9 (30) 6 (20) 4 (13)
CD phenotype, *n* (%) Nonstricturing/nonpenetrating Stricturing Penetrating	14 (47) 1 (3) 2 (7)
History of prior surgery, *n* (%)	0 (0)
History of prior TNF antagonist therapy, *n* (%)	0 (0)
Hospitalization during infliximab start, *n* (%)	17 (57)
Use of concomitant corticosteroids at baseline, *n* (%)	12 (40)
Use of concomitant immunosuppressants at baseline, *n* (%) MTX 6 MP or AZA	13 (43) 9 4
Baseline disease activity index PUCAI, median (IQR) wPCDAI, median (IQR)	50 (30–70) 41 (30–62)
Baseline albumin, g/L, median (IQR)	4 (3.05–4.6)
Baseline CRP, mg/L, median (IQR)	1 (0.25–4.35)

**Table 2:** Patient outcomes.

Full analysis set, *n*	30
Clinical response, *n* (%)	19/30 (63)
Clinical remission, *n* (%)	10/30 (33)
Biochemical response, *n* (%)	15/26 (58)
Biochemical remission, *n* (%)	8/25 (32)
Primary remission, *n* (%)	6/29 (20)
Durability of infliximab use, *n* (%)	25/30 (83)

**Table 3:** Final model parameters.

Final Model Parameter	Final Parameter Estimates (SE)	Median Bootstrap ^a^ Estimates (95% CI)
Θ1 (V_C_; L)	3.43 (0.224)	3.33 (1.05, 3.86)
Θ2 (CL; L/day)	0.252 (0.0244)	0.252 (0.205, 0.307)
Θ3 (V_P_; L)	2.11 (0.403)	2.20 (1.31, 5.05)
Θ4 (Q; L/day)	1.41 (0.438)	1.46 (0.66, 15.21)
Θ5 (Albumin on CL)	−1.73 (0.31)	−1.73 (−2.52, −1.11)
Θ6 (Albumin on VP)	−1.86 (0.27)	−1.87 (−2.60, −0.279)
Θ7 (Albumin on VC)	−0.906 (0.27)	−0.914 (−3.82, −0.443)
**Between Participant Variability**		
IIV on CL	45.4% (7.6%)	43.7% (28.1, 60.3)
IIV on VC	21.6% (6.3%)	20.7% (0.1, 48)
**Error**		
Proportional (%)	26.9% (3.3%)	26.2% (20.1, 33.0)
CL (L/day) = 0.252 × (WT/51.2)^0.75^ × (Albumin/4.2)^−1.73.^ V_C_ (L) = 2.47 × (WT/51.2) × (Albumin/4.2)^−0.906^ V_p_ (L) = 2.11 × (WT/51.2) × (Albumin/4.2)^−1.86^ Q (L/day) = 1.41 × (WT/51.2)^0.75^		

CI, confidence interval; CL, clearance; Q, bioavailability; Q, intercompartmental clearance; SE, standard error; V_C_, volume of distribution of central compartment; V_p_, volume of distribution of peripheral compartment. ^a^ Bootstrap successfully converged 99.6% of the time.

**Table 4:** Overview of infliximab population PK models in pediatric IBD.

Study/Reference	Population	Parameter Estimates	Relationship of Covariate to Pharmacokinetic Parameter
Fasanmade et al. [10]	Pediatric CD (age range 6–17 years) *n* = 112	CL 0.23 L/day Vc 2.28 L Vp 1.23 L Q 0.15 L/day	ALB on CL [−] WT on CL [−] WT on Vc [−] WT on Vp [−]
Bauman et al. [11]	Pediatric CD, UC, IBD-U *n* = 228	CL 0.29 L/day Vc 3.52 L Vp 1.9 L Q 0.23 L/day	ALB on CL [−] WT on CL [+] ATI on CL [+] ESR on CL [+]
Xu et al. [12]	Pediatric CD, UC, JRA, Kawasaki disease (age range 2 months–17 years) UC *n* = 60 CD *n* = 112 JRA *n* = 117 Kawasaki disease = 16	CL 0.30 L/day Vc 3.33 L Vp 1.14 L Q 0.072 L/day	Not available
Xiong et al. [13]	Pediatric CD *n* = 78	CL 0.33 L/day Vc 2.97 L Vp 2.84 Q 0.228 L/day FIXED	WT on CL [+] ALB on CL [−] ESR on CL [+] ATI on Cl [+] WT on Vc [+] WT on Q [+] WT on Vp [+]

CD: Crohn’s disease, UC: ulcerative colitis, IBD-U: inflammatory bowel disease-unclassified, JRA: juvenile rheumatoid arthritis, ALB: albumin, WT: weight, ATI: antibodies to infliximab, ESR: erythrocyte sedimentation rate, CL: clearance, Vc: volume of distribution, Vp: volume of distribution in the peripheral compartment, Q: intercompartmental clearance, [+] indicates a positive correlation, [−] indicates a negative correlation.

## Data Availability

The datasets used and analyzed in this current study are available from the corresponding author on reasonable request.

## Abbreviations

The following abbreviations are used in this manuscript:

BMI	body mass index
CCC	concordance correlation coefficient
CD	Crohn’s disease
CL	clearance
CRP	C-reactive protein
ECLIA	electrochemiluminescence-based immunoassay
ELISA	enzyme-linked immunosorbent assay
ESR	erythrocyte sedimentation rate
HMSA	homogeneous mobility shift assay
IBD	inflammatory bowel disease
IBD-U	inflammatory bowel disease—unspecified
IQR	interquartile range
OFV	objective function value
PK	pharmacokinetic
POC	point-of-care
PUCAI	pediatric ulcerative colitis activity index
Q	intercompartmental clearance
UC	ulcerative colitis
Vc	central compartment volume of distribution
Vp	compartment volume of distribution
wPCDAI	pediatric Crohn’s disease activity index
WBC	White-blood-cell count

## References

[1] Hyams J., Damaraju L., Blank M., Johanns J., Guzzo C., Winter H.S., Kugathasan S., Cohen S., Markowitz J., Escher J.C. (2012). Induction and maintenance therapy with infliximab for children with moderate to severe ulcerative colitis. Clin. Gastroenterol. Hepatol..

[2] Hyams J., Crandall W., Kugathasan S., Griffiths A., Olson A., Johanns J., Liu G., Travers S., Heuschkel R., Markowitz J. (2007). Induction and maintenance infliximab therapy for the treatment of moderate-to-severe Crohn’s disease in children. Gastroenterology.

[3] Naviglio S., Lacorte D., Lucafò M., Cifù A., Favretto D., Cuzzoni E., Silvestri T., Mucelli M.P., Radillo O., Decorti G. (2019). Causes of treatment failure in children with inflammatory bowel disease treated with infliximab: A pharmacokinetic study. J. Pediatr. Gastroenterol. Nutr..

[4] Vande Casteele N., Jeyarajah J., Jairath V., Feagan B.G., Sandborn J.W. (2019). Infliximab exposure-response relationship and thresholds associated with endoscopic healing in patients with ulcerative colitis. Clin. Gastroenterol. Hepatol..

[5] van Hoeve K., Seyed Tabib N.S., Dreesen E., Tops S., Hoffman I., Gils A., Ferrante M., Vermeire S. (2022). Infliximab concentrations during induction are predictive for endoscopic remission in pediatric patients with inflammatory bowel disease under combination therapy. J. Pediatr..

[6] Papamichael K., Vande Casteele N., Jeyarajah J., Jairath V., Osterman M.T., Cheifetz A.S. (2021). Higher Postinduction Infliximab Concentrations Are Associated with Improved Clinical Outcomes in Fistulizing Crohn’s Disease: An ACCENT-II Post Hoc Analysis. Am. J. Gastroenterol..

[7] Stenke E., Alhassan D., Moclair M., Cooper S., Dominika A., Lang C., Quinn S., Broderick A., Fitzpatrick E., Bourke B. (2025). Higher-Dose Infliximab Induction Achieves Better Maintenance Trough Levels in a National Pediatric IBD Cohort-A Retrospective Study. Inflamm. Bowel Dis..

[8] Hoelz H., Bragagna L., Litwin A., Koletzko S., Le Thi T.G., Schwerd T. (2024). Pediatric IBD Patients Treated with Infliximab and Proactive Drug Monitoring Benefit from Early Concomitant Immunomodulatory Therapy: A Retrospective Analysis of a 10-Year Real-Life Cohort. Inflamm. Bowel Dis..

[9] Roberts J.K., Stockmann C., Balch A., Yu T., Ward R.M., Spigarelli M.G., Sherwin C.M.T. (2015). Optimal design in pediatric pharmacokinetic and pharmacodynamic clinical studies. Pediatr. Anesth..

[10] Fasanmade A.A., Adedokun O.J., Blank M., Zhou H., Davis H.M. (2011). Pharmacokinetic properties of infliximab in children and adults with Crohn’s disease: A retrospective analysis of data from 2 phase III clinical trials. Clin. Ther..

[11] Bauman L.E., Xiong Y., Mizuno T., Minar P., Fukuda T., Dong M., Rosen M.J., Vinks A.A. (2020). Improved Population Pharmacokinetic Model for Predicting Optimized Infliximab Exposure in Pediatric Inflammatory Bowel Disease. Inflamm. Bowel Dis..

[12] Xu Z., Mould D., Hu C., Ford J., Keen M., David H., Zhou H. Population Pharmacokinetic Analysis of Infliximab in Pediatrics Using Integrated Data from Six Clinical Trials [abstract]. Proceedings of the American College of Clinical Pharmacology Annual Meeting.

[13] Xiong Y., Mizuno T., Colman R., Hyams J., Noe J.D., Boyle B., Tsai Y.T., Dong M., Jackson K., Punt N. (2021). Real-World Infliximab Pharmacokinetic Study Informs an Electronic Health Record-Embedded Dashboard to Guide Precision Dosing in Children with Crohn’s Disease. Clin. Pharmacol. Ther..

[14] Singh N., Rosenthal C.J., Melmed G.Y., Mirocha J., Farrior S., Callejas S., Tripuraneni B., Rabizadeh S., Dubinsky M.C. (2014). Early infliximab trough levels are associated with persistent remission in pediatric patients with inflammatory bowel disease. Inflamm. Bowel Dis..

[15] Lawrence S., Faytrouni F., Harris R.E.M., Irvine M., Carrion E., Scott G., Clarke B., Garrick V.M., Curtis L.B., Gervais L.B. (2022). Optimized Infliximab Induction Predicts Better Long-Term Clinical and Biomarker Outcomes Compared to Standard Induction Dosing. J. Pediatr. Gastroenterol. Nutr..

